# High temperature influences DNA methylation and transcriptional profiles in sea urchins (*Strongylocentrotus intermedius*)

**DOI:** 10.1186/s12864-023-09616-7

**Published:** 2023-08-28

**Authors:** Anzheng Liu, Fanshuang Zeng, Luo Wang, Hao Zhen, Xinglong Xia, Honglin Pei, Changkun Dong, Yanmin Zhang, Jun Ding

**Affiliations:** Key Laboratory of Mariculture & Stock Enhancement in North China Sea, Ministry of Agriculture and Rural Affairs, Dalian Ocean University, Dalian, 116023 China

**Keywords:** Sea urchin (*Strongylocentrotus intermedius*), High temperature, Epigenetic, Methylation, Transcriptome

## Abstract

**Background:**

DNA methylation plays an important role in life processes by affecting gene expression, but it is still unclear how DNA methylation is controlled and how it regulates gene transcription under high temperature stress conditions in *Strongylocentrotus intermedius*. The potential link between DNA methylation variation and gene expression changes in response to heat stress in *S. intermedius* was investigated by MethylRAD-seq and RNA-seq analysis. We screened DNA methylation driver genes in order to comprehensively elucidate the regulatory mechanism of its high temperature adaptation at the DNA/RNA level.

**Results:**

The results revealed that high temperature stress significantly affected not only the DNA methylation and transcriptome levels of *S. intermedius* (*P* < 0.05), but also growth. MethylRAD-seq analysis revealed 12,129 CG differential methylation sites and 966 CWG differential methylation sites, and identified a total of 189 differentially CG methylated genes and 148 differentially CWG methylated genes. Based on KEGG enrichment analysis, differentially expressed genes (DEGs) are mostly enriched in energy and cell division, immune, and neurological damage pathways. Further RNA-seq analysis identified a total of 1968 DEGs, of which 813 genes were upregulated and 1155 genes were downregulated. Based on the joint MethylRAD-seq and RNA-seq analysis, metabolic processes such as glycosaminoglycan degradation, oxidative phosphorylation, apoptosis, glutathione metabolism, thermogenesis, and lysosomes are regulated by DNA methylation.

**Conclusions:**

High temperature affected the DNA methylation and expression levels of genes such as *MOAP-1*, *GGT1* and *RDH8*, which in turn affects the metabolism of HPSE, Cox, glutathione, and retinol, thereby suppressing the immune, energy metabolism, and antioxidant functions of the organism and finally manifesting as stunted growth. In summary, the observations in the present study improve our understanding of the molecular mechanism of the response to high temperature stress in sea urchin.

## Background

Global ocean warming has imposed environmental stress on many marine organisms [1–3]. Over the past century, the development, survival, growth, and metabolism of many organisms have been affected by high temperatures [4, 5]. However, organisms possess mechanisms to buffer the negative impact of high temperatures on their development and physiology. These mechanisms to withstand increased temperatures, such as the heat shock response [6], developmental arrest [7], and behavioral adaptation [8], may be a key determinant of a species’ future health and persistence. Although many buffering mechanisms (such as heat shock gene regulation) have been highly conserved over long periods of evolution [9], there may be substantial differences in tolerance to higher temperatures among related species [10]. This suggests that temperature tolerance can evolve rapidly. The mechanisms of stress responses and evolution can be directly linked: the way a species responds to stress not only determines its current level of tolerance, but may also evolve into higher tolerance by affecting the expression of different genes [11]. This phenomenon is referred to as epigenetic regulation, involving multiple mechanisms, with DNA methylation being one of the most extensively studied and important mechanisms [12].

During stress episodes, such as high temperature, genomic DNA methylation regulates resistance by influencing gene expression [13]. A growing body of evidence supports the crucial role of epigenetic regulatory mechanisms in the adaptive response of organisms to environmental stress [14]. For example, studies have shown that in *Dicentrarchus labrax*, elevated temperature was found to significantly alter its DNA methylation pattern and the expression of genes associated with DNA methylation, the stress response, and muscle and organ formation [15]. In *Salmo salar*, high temperature stress has been shown to induce methylation changes at multiple CpG sites in various genomic elements surrounding the transcription start sites (TSSs) of *cirbp*, *serpinh1*, *prdx6*, *ucp2*, and *jund* [16]. Feeding temperature was found to influence promoter methylation levels in the skeletal muscle of *Solea senegalensis* Kaup. At lower temperatures (15℃), elevated myog methylation levels led to the downregulation of myog transcription compared to higher feeding temperatures (18℃ and 21℃). Changes in myog methylation levels affected organism growth and muscle cell structure [17].

Promoter DNA methylation is an important regulatory component of gene expression, which has been well studied in vertebrates [18], however, its role in gene regulation in invertebrates has rarely been studied [19]. Recent studies have shown that promoter methylation also plays an important role in invertebrates [20–22]. Earlier studies have shown that DNA methylation in gene promoter regions can repress gene transcription and expression [23], but the association between DNA methylation and gene expression is not simple. The diversification of relationships between different genes, cell types, and promoter methylation may lead to the organism exhibiting complex adaptive mechanisms in the face of environmental stress [24]. The findings of single-omics studies are often limited, and the integration of multi-omics analysis offers novel insights to decipher the complex biological mechanisms of epigenetic regulation. In chronic stress experiments on juvenile Atlantic salmon (cold shock during embryonic development), combined transcriptomic and methylomic analyses showed a considerable effect of early life stress on immune competence and disease susceptibility [25]. In triploid sea cucumber (*Apostichopus japonicus*) body wall tissue, a combined transcriptomic and methylomic analysis revealed a total of 19 co-expressed genes, which were mainly enriched in metabolic processes such as gluconeogenesis, lipid metabolism, and histidine metabolism. These pathways play an important role in growth and development [26].

*Strongylocentrotus intermedius* is a representative echinoderm species and it serves as a model organism in studies of embryonic development [27]. In recent years, due to global ocean warming, sea water temperatures in coastal intertidal zones have often been higher than 25 °C (the lethal limit of *S. intermedius*) in the summer, resulting in the massive death of cultured *S. intermedius* [28, 29]. Studies on the molecular regulation of the response to high temperature stress in *S. intermedius* have mainly focused on the RNA level [27, 30]; studies on the epigenetic regulation of the response to high temperature stress in *S. intermedius* at the DNA level have not been reported.

Therefore, in this study, we used polynomial fitting and response surface methodology (RSM) to (i) establish a prediction model for the growth of sea urchins with different shell diameters under different temperature conditions and (ii) determine the optimal temperature and limit temperature for the growth of sea urchins with different shell diameters. In addition, we constructed a DNA methylation library of the gut wall of *S. intermedius* at high temperature and moderate temperature was constructed by MethylRAD-seq and RNA-seq analysis. We screened for heat stress response-related genes and regulatory pathways and identified potential links between promoter DNA methylation variation and gene expression changes in *S. intermedius* in response to high temperature stress, The aim of this was to comprehensively elucidate the regulatory mechanisms of its high temperature adaptation at the DNA/RNA level and providing a theoretical basis for the breeding of new varieties.

## Results

### Effect of temperature on the growth of *S. intermedius*

#### Weight gain rate

The Weight gain rate (WGR) of *S. intermedius* with different shell diameters at different temperatures were investigated. The WGR of *S. intermedius* with shell diameters of 2 cm, 4 cm, and 6 cm was highest in the 14 °C group (5.60%, 4.76%, and 1.97%, respectively). At 26 °C, the weight of *S. intermedius* with different shell diameters decreased.

#### Construction of growth prediction model for *S. intermedius*

Using RSM, the model equation of WGR of *S. intermedius* with different shell diameters at different temperatures was found to be as follows: WGR (%) = -6.1175 + 0.2177 × *D* + 1.5362 × *T* + 0.0054 × *DT* − 0.1414 × *D*^2^ − 0.0511 × *T*^2^, where *D* is the middle spherical shell diameter of the sea urchin (cm) and *T* is the temperature of the aquaculture water (°C).

The regression model established is very significant (*P* < 0.01), indicating that the equation is effective. At the same time, the *R*^2^ value of the model equation is 0.9369, indicating that the model can explain 93.69% of the change in response value.

**Fig. 1:**
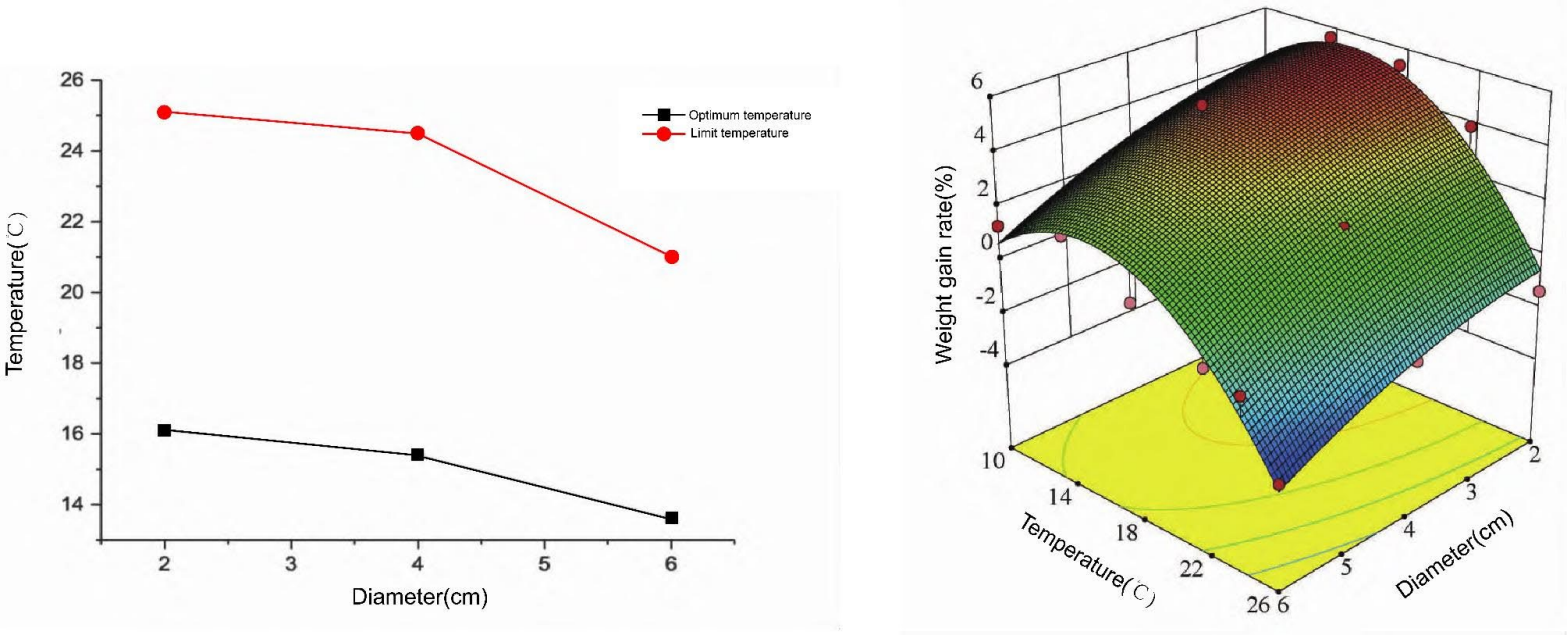
Growth of *Strongylocentrotus intermedius* with different shell diameters at different temperatures. **(A)** The optimum and extreme temperature for growth of *S. intermedius* with different shell diameter. **(B)** Response surface plot of temperature, diameter and their interaction in growth of *S. intermedius*

The optimal temperature and limit temperature calculated by the model are shown in Fig. 1. The optimal growth temperature of *S. intermedius* with a shell diameter of 2 cm is 16.1 °C, and the growth limit temperature is 25.1 °C; the optimal growth temperature of *S. intermedius* with a shell diameter of 4 cm is 15.4 °C, and the growth limit temperature is 24.5 °C; and the optimal growth temperature of *S. intermedius* with a shell diameter of 6 cm is 13.6 °C, and the growth limit temperature is 21.0 °C.

#### Model validation

The prediction model for the growth of *S. intermedius* with different shell diameters at different temperatures established in this study has a good fit. The response surface model was used to optimize the experimental conditions. We found that the maximum WGR of *S. intermedius* with shell diameters of 2 cm, 4 cm, and 6 cm at the optimal growth temperature is 5.47%, 4.36%, and 2.14%, respectively. To verify the reliability of the model, 300 sea urchins were used to conduct validation experiments according to the predicted optimal conditions. The measured WGRs of *S. intermedius* were 5.73%, 4.04%, and 1.87%, which were consistent with the theoretical values, indicating that the model is reasonable and effective.

### DNA methylation analysis

#### DNA methylation sequencing analysis

MethylRAD sequencing and data analysis were performed. The results showed that an average of 28,976,363 raw reads were obtained for each sample. After filtering, the clean reads (Table 1) were compared to the reference genome. The proportion of clean reads that could be mapped to a unique position of the reference sequence ranged from 80 to 82%.

**Table 1:** Sample sequencing data volume and comparison rate

Group	Sample	Raw Reads	Clean Reads	Percent	Uniquely mapped reads	Multiple mapped reads	Mapped Ratio
MD	MD1	31,019,665	14,801,913	73.97%	5,992,389	5,993,565	80.98%
MD	MD2	34,096,563	15,535,383	76.25%	6,169,774	6,512,609	81.64%
MD	MD3	30,876,782	15,519,001	76.24%	6,205,494	6,367,305	81.02%
MG	MG1	24,312,147	14,557,317	72.52%	5,930,858	5,825,361	80.76%
MG	MG2	30,124,811	15,217,314	74.58%	6,112,333	6,180,455	80.78%
MG	MG3	23,428,212	14,448,674	71.56%	5,865,613	5,840,737	81.02%

#### The distribution of methylation sites

**Fig. 2:**
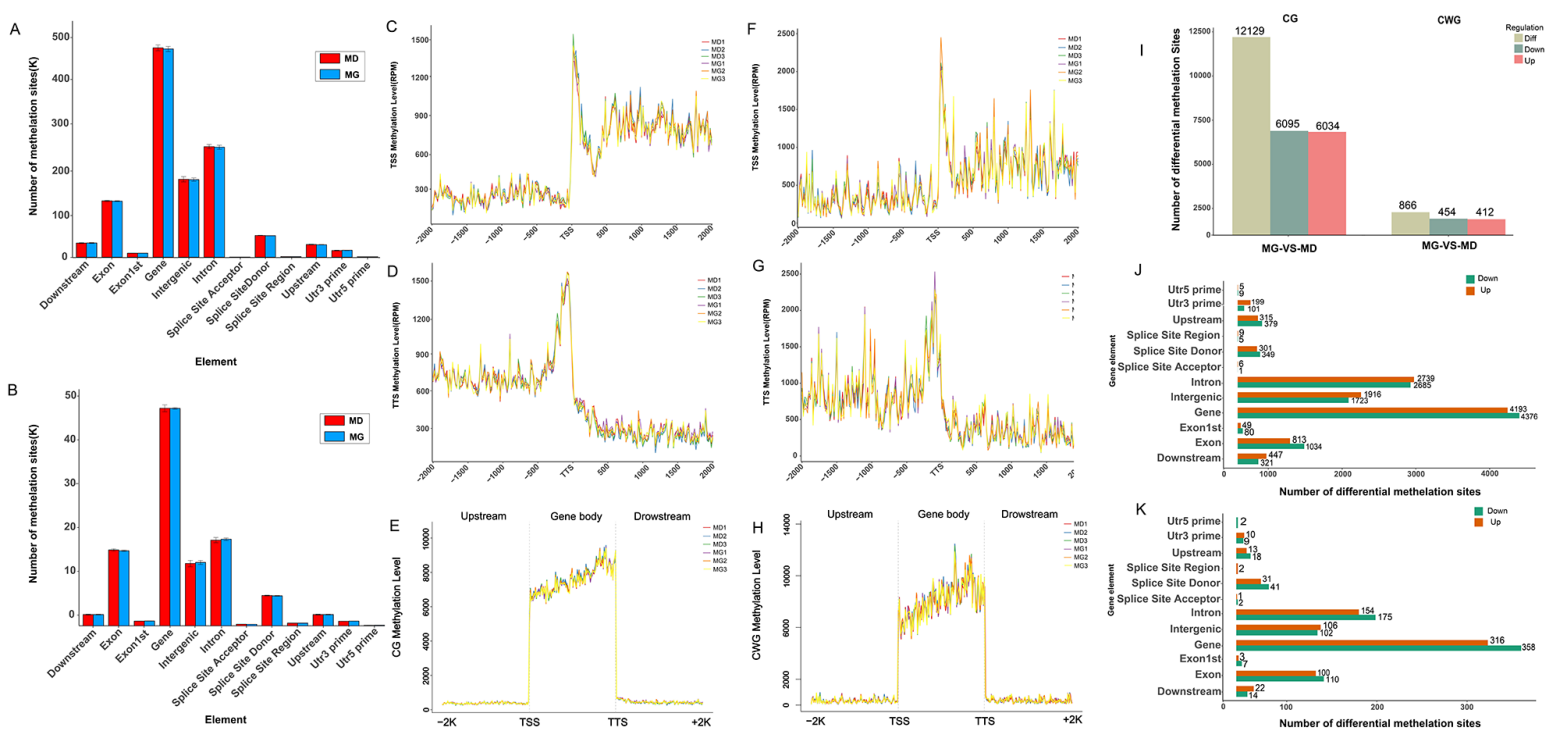
Statistical analysis of methylation sites in *Strongylocentrotus intermedius* under high temperature stress. **(A)** Distribution of CG methylation sites on different gene functional elements. **(B)** Distribution of CWG methylation sites on different gene functional elements. **(C)** Distribution of CG sites in TSSs. **(D)** Distribution of CG sites in TTSs. **(E)** Distribution of CG sites in TSSs, TTSs, and gene bodies. **(F)** Distribution of CWG sites in TSSs. **(G)** Distribution of CWG sites in TTSs. **(H)** Distribution of CWG sites in TSSs, TTSs, and gene bodies. **(I)** Distribution of CG differential methylation sites on different gene functional elements. **(J)** Distribution of CWG differential methylation sites on different gene functional elements. **(K)** Statistical analysis of differential methylation sites

According to the results of reference genome mapping, the number of methylation sites screened in the samples and the average sequencing depth were determined (Table 2). The average number of CG methylation sites in control sea urchins was 684,527, and the average number of CG methylation sites in high temperature sea urchins was 680,788; the average number of CWG methylation sites in control sea urchins was 60,908, and the average number of CWG methylation sites in high temperature sea urchins was 61,157. According to the annotation based on the location information of methylation sites, the CG and CWG methylation sites were distributed differently on different functional elements, but the distribution trends of both CG and CWG sites were similar, with the highest proportion of methylation sites distributed in the gene body, followed by the intron region (Fig. 2A and B). The reads in the 2-kb regions upstream of TSSs, gene bodies, and 2-kb regions downstream of TTSs were counted. The DNA methylation levels were similar among the samples, and the DNA methylation sites were mostly distributed in the gene bodies; the DNA methylation site distribution curve was significantly higher in the regions upstream of TSSs and downstream of TTSs than in other sequences (Fig. 2C-H).

**Table 2:** Statistics of methylation site data and depth

Group	Sample	CG site number	CG site depth	CWG site number	CWG site depth
MD	MD1	690,098	9.81	61,538	7.42
MD	MD2	669,447	10.62	59,302	8.36
MD	MD3	694,039	10.22	61,884	7.99
MG	MG1	691,589	9.69	61,715	7.35
MG	MG2	677,928	10.31	60,860	7.86
MG	MG3	672,846	9.89	60,896	7.50

After normalization of the sequencing depth information, 12,129 CG differentially methylated sites (CG-DMSs) and 966 CWG differentially methylated sites (CWG-DMSs) were screened (Fig. 2I). The distribution of DMSs on different functional elements is detailed in Fig. 2J and K.

#### Enrichment analysis of DMS-related genes

**Fig. 3:**
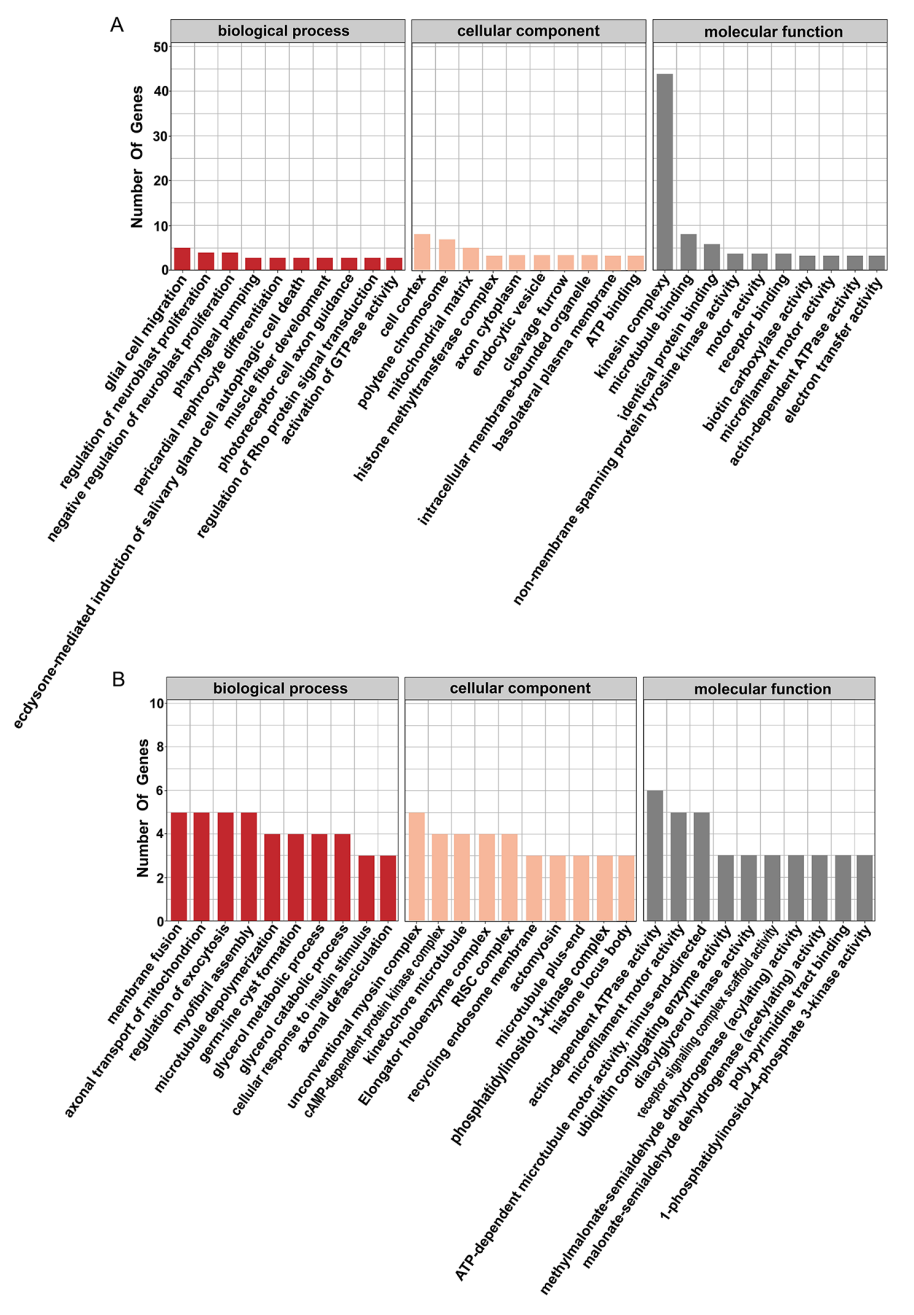
GO Enrichment analysis of differentially methylated genes at the site level of Strongylocentrotus intermedius under high temperature stress. **(A)** GO enrichment analysis of the top 30 of CWG differentially methylated genes at the site level. **(B)** GO enrichment analysis of the top 30 of CG differentially methylated genes at the site level

GO functional enrichment analysis showed that in CG-DMS-related genes, significantly enriched GO terms included protein phosphorylation, cytoplasm, and ATP binding (Fig. 3A). The most significantly enriched GO terms in CWG-DMS-related genes included intracellular signal transduction, cell cortex, and ATP binding (Fig. 3B).

**Fig. 4:**
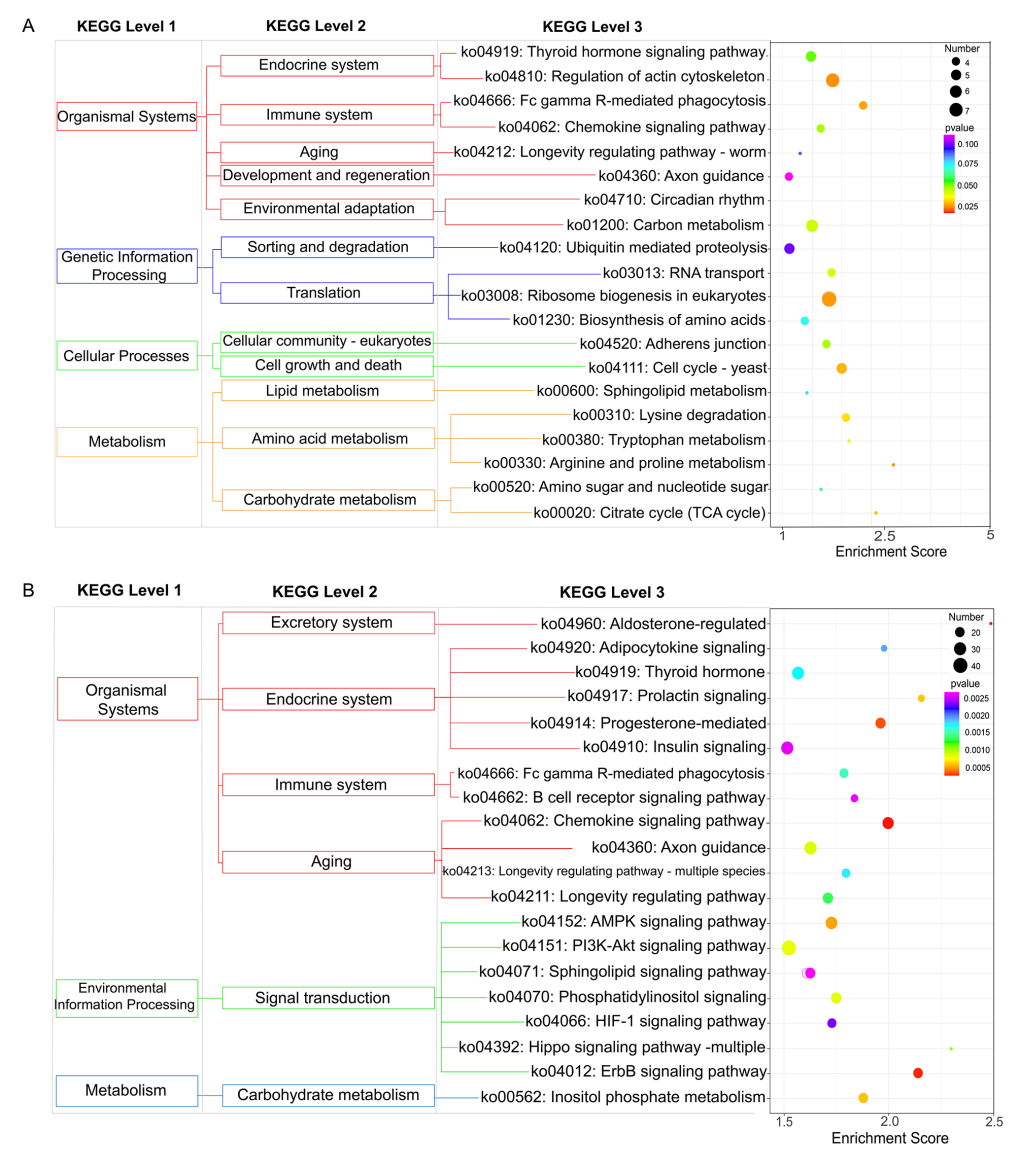
KEGG Enrichment analysis of differentially methylated genes at the site level of Strongylocentrotus intermedius under high temperature stress. **(A)** KEGG enrichment analysis of the top 20 of CWG differentially methylated genes at the site level. **(B)** KEGG enrichment analysis of the top 20 of CG differentially methylated genes at the site level

KEGG pathway enrichment analysis was conducted to screen the biological pathways of DMS-related genes. Figure 4 A shows the top 20 KEGG pathways in which CG-DMS-related genes are enriched. DMS-related genes were significantly enriched in aldosterone-regulated sodium reabsorption, ErbB signaling, and AMPK signaling. Figure 4B shows the top 20 KEGG pathways enriched in CWG-DMS-related genes, including pathways such as regulation of the actin cytoskeleton, RNA transport, and arginine and proline metabolism.

#### Analysis of methylation differences at the gene level

**Fig. 5:**
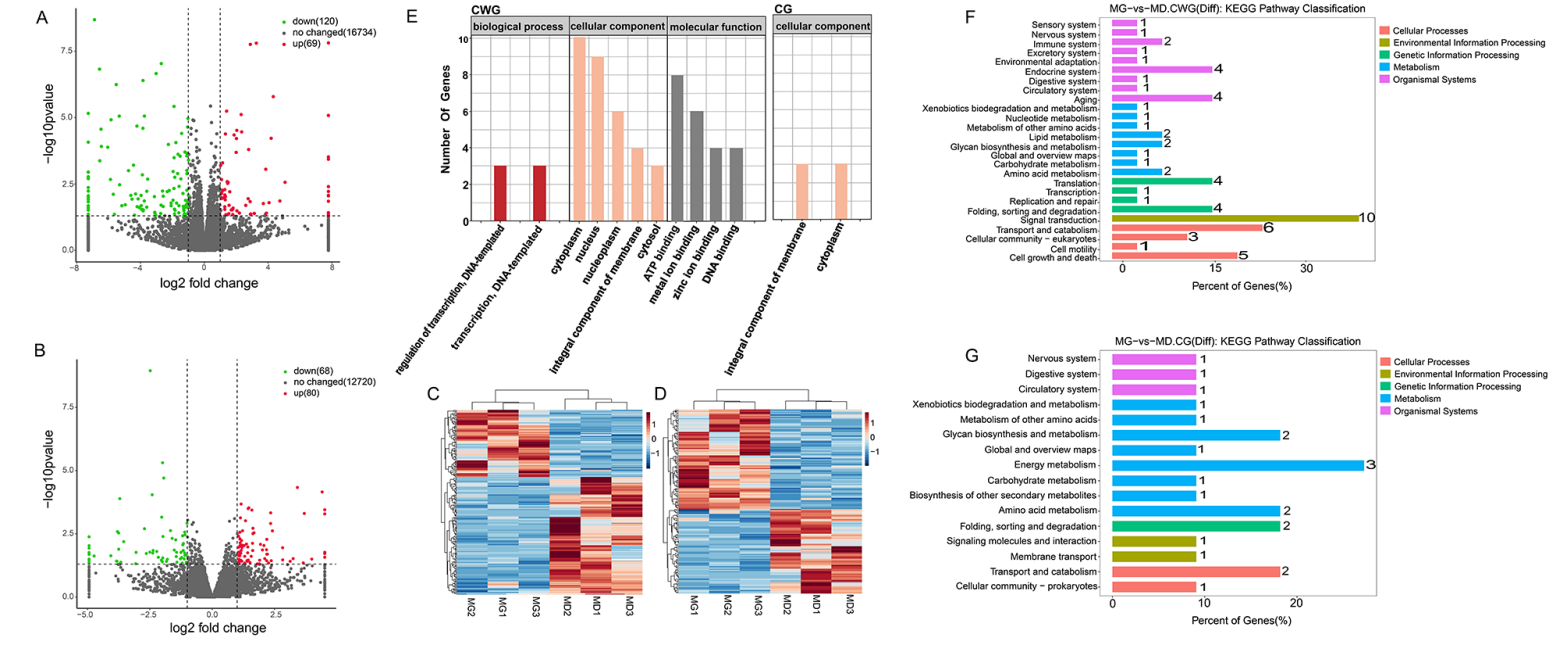
Analysis of methylation differences at the gene level in Strongylocentrotus intermedius under high temperature stress. **(A)** Volcano plot of differential expression of genes methylated at CG sites. **(B)** Volcano plot of differential expression of genes methylated at CWG sites. **(C)** Heatmap of differential methylation gene clustering among CG sites. **(D)** Heatmap of differential methylation gene clustering among CWG sites. **(E)** Bar chart of GO functional classification of differentially methylated genes. **(F)** KEGG pathway enrichment analysis of CG differentially methylated genes. **(G)** KEGG pathway enrichment analysis of CWG differentially methylated genes

The sum of all methylation site levels within a single gene was used to represent the methylation level of that gene, and genes with *P* < 0.05 and |log_2_(fold change)| > 1 were screened. A total of 189 differentially methylated genes (DMGs) were found for CG between the two groups, of which 69 were upregulated and 120 were downregulated, and 148 DMGs were found for CWG, of which 80 were upregulated and 68 were downregulated (Fig. 5A and B). The DMGs were clustered and a heatmap was generated (Fig. 5C and D).

DMGs were GO-annotated and GO terms with more than two DMGs were screened for biological processes, cell composition, and molecular function (Fig. 5E). Among them, CG differentially methylated genes were not significantly enriched in GO functions. CWG differentially methylated genes were enriched in transcriptional regulatory DNA templates and transcriptional DNA templates in the biological process category; in nucleoplasmic and cytoplasmic processes and membrane components in the cellular component category; and in zinc ion binding, DNA binding, and ATP binding in the molecular function category, where zinc ion binding and ATP-binding methylation were upregulated.

KEGG pathway enrichment analysis showed that the CG DMGs were significantly enriched in arginine and proline metabolism, glycine, serine, and threonine metabolism, and oxidative phosphorylation, among which the methylation levels of arginine and proline metabolism and glycine, serine, and threonine metabolism genes were significantly downregulated; the CWG DMGs were significantly enriched in RNA transport, apoptosis, and cell cycle, among which methylation was significantly upregulated in RNA transport and endocytosis genes (Fig. 5F and G).

### Transcriptome analysis

#### Transcriptome sequencing and screening of differential genes

**Fig. 6:**
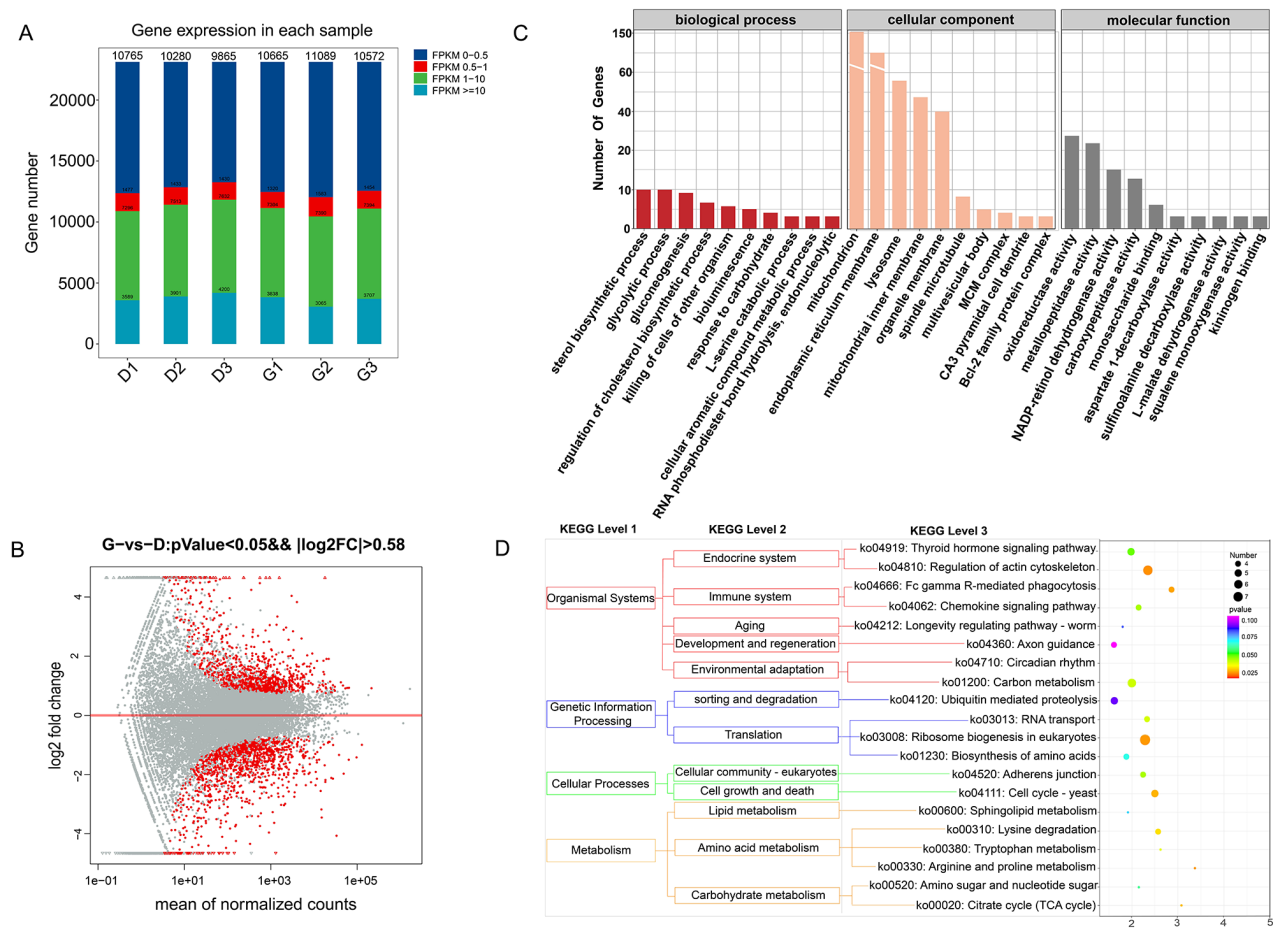
Transcriptome analysis of Strongylocentrotus intermedius under high temperature stress. **(A)** FPKM distribution. **(B)** MA plot of differential expression of genes. **(C)** GO enrichment analysis of the top 30 differentially expressed genes. **(D)** KEGG pathway enrichment analysis of the top 20 differentially expressed genes

High throughput sequencing generated 142.59 Mb and 138.73 Mb of raw reads in the control and high temperature treatment groups, respectively, and after filtering, 141.93 Mb and 138.11 Mb of clean reads were obtained. In the control group, Q30 ≥ 95.87%, and in the high temperature group, Q30 ≥ 95.94% (Table 3). Gene expression levels were expressed as FPKM values; the FPKM values were 24.02, 23.36, and 21.61 in the control group and 22.85, 25.96, and 23.56 in the experimental group. There was no significant effect of high temperature on the number of expressed genes in 4 cm shell sea urchins (Fig. 6A). A total of 23,127 expressed genes were detected by transcriptome sequencing analysis. Statistical analysis (FDR < 0.05 and |log_2_(fold change)| > 1) was performed on the detected expressed genes, and the number of differentially expressed genes (DEGs) detected was 1968, with 813 genes significantly upregulated and 1155 genes significantly downregulated compared to the control group (Fig. 6B).

**Table 3:** Quality assessment of transcriptome sequencing data

Sample	RawReads	RawBases	CleanReads	CleanBases	ValidBases	Q30	GC
D1	47.31 M	7.10G	47.09 M	7.01G	98.78%	95.87%	42.15%
D2	47.74 M	7.16G	47.52 M	7.07G	98.77%	95.97%	42.63%
D3	47.54 M	7.13G	47.32 M	7.04G	98.67%	96.10%	44.93%
G1	47.31 M	7.10G	47.10 M	7.01G	98.79%	96.04%	43.33%
G2	43.76 M	6.56G	43.58 M	6.49G	98.89%	95.94%	40.76%
G3	47.66 M	7.15G	47.43 M	7.06G	98.75%	95.99%	42.10%

#### GO and KEGG enrichment analysis

The top 30 enriched GO terms are shown in Fig. 6C. The results show that the most significantly enriched GO term in the biological process category is cellular amino acid biosynthesis, the most significantly enriched GO term in the cellular component category is the organelle membrane, and the most significantly enriched GO term in the molecular function category is aspartate 1-decarboxylase activity.

The KEGG enrichment analysis results showed that 1968 DEGs were enriched in a total of 328 tertiary metabolic pathways, among which 78 tertiary metabolic pathways were significantly differentially expressed (*P* < 0.05). The pathways with more than two DEGs were screened and sorted according to the − log_10_(*P*-value) corresponding to each entry in descending order (Fig. 6D). DEGs were significantly enriched in signaling pathways such as glyoxylate and dicarboxylic acid metabolism, glycolysis/gluconeogenesis, endoplasmic reticulum protein processing, cytochrome P450 metabolism of foreign factors, steroid synthesis, and glutathione metabolism. Upregulated DEGs were mainly involved in the MAPK signaling pathway and endoplasmic reticulum protein processing; downregulated genes were mainly enriched in cytochrome P450 metabolism of foreign factors, glyoxylate and dicarboxylic acid metabolism, glycolysis/gluconeogenesis, and other pathways.

### Conjoint methylome and transcriptome analysis

**Fig. 7:**
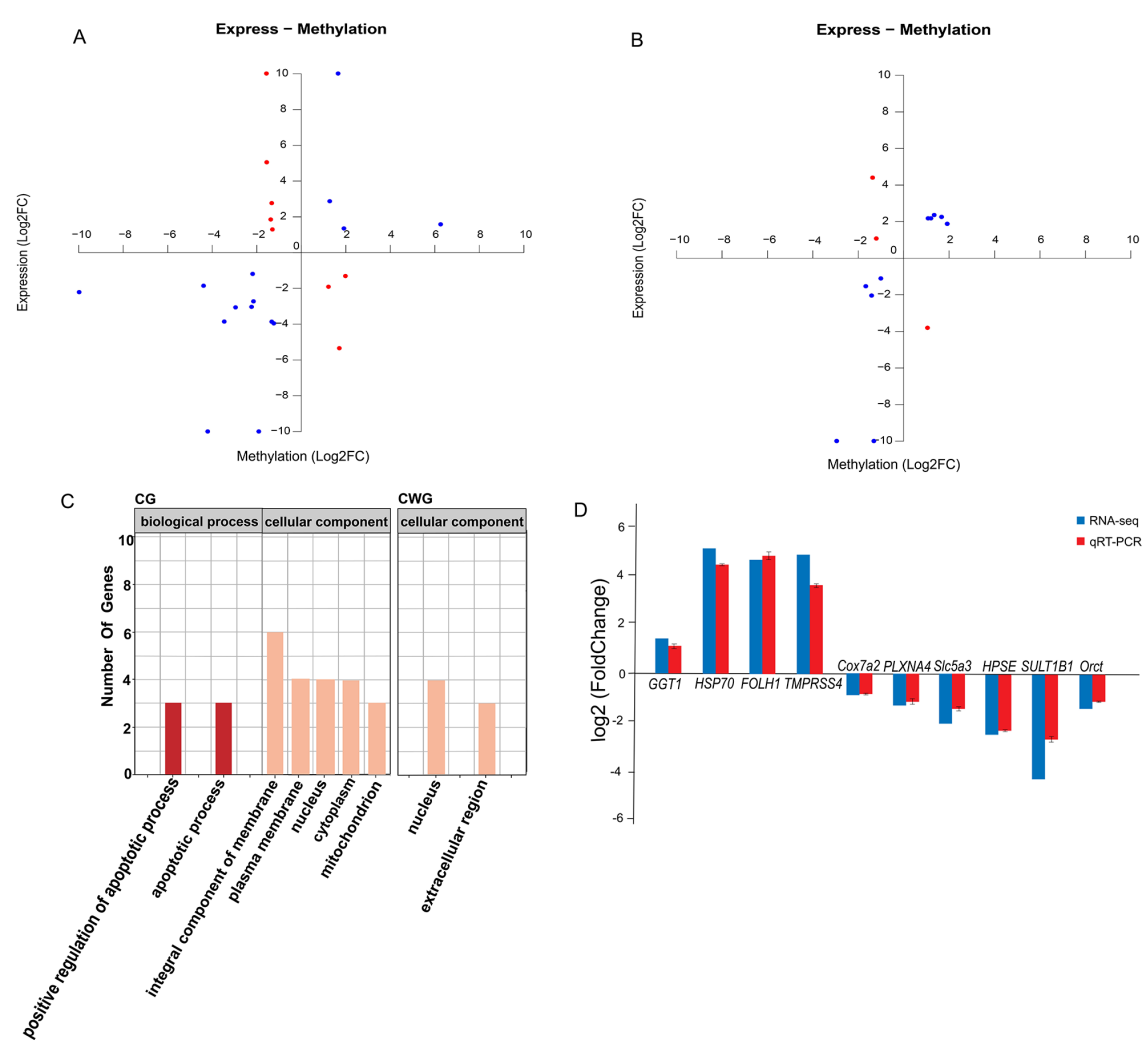
Combined DNA methylation and transcriptome analysis of *Strongylocentrotus intermedius* under high temperature stress. **(A)** Quadrant analysis of differentially expressed genes and CG differentially methylated genes. **(B)** Quadrant analysis of differentially expressed genes and CWG differentially methylated genes. Red indicates negatively associated loci of differential genes, and blue indicates positively associated loci of differential genes. **(C)** GO enrichment analysis of DMGs which were also DEGs. **(D)** Verification of 10 randomly selected DEGs by qRT-PCR.

Conjoint analysis of DMGs in promoter regions and DEGs of 4 cm shell *S. intermedius* under high temperature stress were performed. In total, 23 genes were screened, namely *PLXNA4*, *ft*, *Cox7a2*, *cah-3*, *Slc5a3*, *MKX*, *Orct*, *Moap1*, *pats1*, *HPSE*, *SULT1B1*, *bbs5*, *FAXDC2*, *Ids*, *GGT1*, *tll1*, *Kansl1*, *RBM34*, *SMCHD1*, *RDH8*, *PRADC1*, *Ighmbp2*, and *Hint2*, where *Ids* and *HPSE* were screened simultaneously in both models. In addition, 15 genes were screened based on differential methylation at CG loci, with 8 positively and 7 negatively associated genes (*PLXNA4*, *ft*, *Cox7a2*, *MKX*, *pats1*, *HPSE*, *FAXDC2*), and 10 genes were screened based on differential methylation at CWG loci, with 8 positively associated genes and 2 negatively associated genes (*SMCHD1* and *HPSE*) (Fig. 7A and B).

GO enrichment analysis was performed for co-expressed genes. The results showed that 15 related genes screened based on differential expression at CG loci were enriched in 7 GO terms and were significant (*P* < 0.05) in 2 GO terms, including positive regulation of apoptotic process, apoptotic process, mitochondria, membrane components, plasma membrane, nucleus, and cytoplasm. The 10 genes screened based on differential expression at CWG loci were enriched in 2 GO terms and significant (*P* ≤ 0.05) in 1 GO term, including extracellular region and nucleus (Fig. 7C).

KEGG enrichment analysis showed that genes with differential methylation at CG loci were significantly enriched in 16 related pathways (*P* < 0.05), including taurine and hypotaurine metabolism, glycosaminoglycan degradation, base excision repair, oxidative phosphorylation, apoptosis, glutathione metabolism, arachidonic acid metabolism, thermogenesis, and lysosomes (Table 4). Genes with differential methylation at CWG loci were significantly enriched in three related pathways (*P <* 0.05), including glycosaminoglycan degradation, retinol metabolism, and lysosomes (Table 5).

**Table 4:** CG enriched to KEGG pathway, differential ploidy and related genes

KEGG: ID	Term	*P* value	Gene ID
ko00430	Taurine and hypotaurine metabolism	0.0000377	SISin21G005860
ko00531	Glycosaminoglycan degradation	0.0002576	SISin17G005750
ko04260	Cardiac muscle contraction	0.0003375	SISin02G005860
ko03410	Base excision repair	0.0003814	SISin01G000750
ko04212	Longevity regulating pathway - worm	0.0015293	SISin01G000750
ko00190	Oxidative phosphorylation	0.0022179	SISin02G005860
ko04210	Apoptosis	0.0023815	SISin01G000750
ko00480	Glutathione metabolism	0.0023815	SISin21G005860
ko04932	Non-alcoholic fatty liver disease (NAFLD)	0.0027256	SISin02G005860
ko05012	Parkinson disease	0.0044729	SISin02G005860
ko00590	Arachidonic acid metabolism	0.0050930	SISin21G005860
ko05131	Shigellosis	0.0067180	SISin18G001790
ko04714	Thermogenesis	0.0076581	SISin02G005860
ko04142	Lysosome	0.0091760	SISin17G005750
ko05016	Huntington disease	0.0122271	SISin02G005860
ko05010	Alzheimer disease	0.0169127	SISin02G005860

**Table 5:** CWG enriched to KEGG pathway, differential ploidy and related genes

KEGG: ID	Term	*P* value	Gene ID
ko00531	Glycosaminoglycan degradation	0.0000260	SISin17G005750
ko00830	Retinol metabolism	0.0004995	SISin09G000480
ko04142	Lysosome	0.0009767	SISin17G005750

### Analytical validation of key genes

Some DEGs were selected for RT-qPCR validation. The results showed that the mRNA levels of *Cox7a2*, *PLXNA4*, *Slc5a3*, *SULT1B1*, *Orct*, and *HPSE* in the high temperature group were lower than those in control urchins; the mRNA levels of *GGT1*, *FOLH1*, *TMPRSS4*, and *HSP70* were higher in the high temperature group than in control sea urchins. The results showed that the qRT-PCR results were consistent with the transcriptome sequencing results (Fig. 7D).

## Discussion

The increase in ocean temperature due to climate change affects the development of *S. intermedius* [31]. High temperature stress is an important external stimulus, which induces a series of heritable changes to the DNA, namely epigenetic changes [32]. Epigenetic mechanisms can enable the body to respond to environmental conditions and establish different functions to cope with environmental stress [33]. In order to comprehensively elucidate the regulatory mechanism of *S. intermedius*’ high temperature adaptation at the DNA/RNA level, we investigated the potential link between DNA methylation variation and gene expression changes in response to heat stress by MethylRAD-seq and RNA-seq analysis.

In order to find the optimal temperature and limit temperature for the growth of *S. intermedius*, we developed a growth prediction model by polynomial fitting and RSM. This study demonstrates a significant correlation between the growth of *S. intermedius* and temperature. The temperature tolerance of *S. intermedius* varies at different stages of development. Similar observations have been made in previous marine biological studies [34–36]. The larger the shell diameter, the lower the optimal temperature and the limit temperature. By constructing a growth model, we determined that the optimal temperature of *S. intermedius* of different sizes were 16.1 °C, 15.4 °C, and 13.6 °C. The validation experiment showed that the model was reliable. Since *S. intermedius* at the mature stage measures 4 cm, the growth and development of various organs is relatively rapid, so we chose the 4 cm shell diameter sea urchin to carry out the molecular mechanism study at high temperature.

DNA methylation, which is one of the most studied and important epigenetic regulatory mechanisms, plays an important role by influencing the expression of genes [37]. MethylRAD-seq is a promising technology that gives very reliable results in aquatic animals [38–40]. In this study, the genomic DNA methylation of *S. intermedius* in the heat stress group and the control group was studied using MethylRAD-seq technology. A large number of methylation sites and differentially methylated genes were obtained. The results showed that each sample had significantly more CG methylation sites than CWG methylation sites. Statistical analysis of the methylation site distribution revealed that the distribution of CG and CWG methylation sites on different functional elements is different, but the distribution trend is similar. Methylation sites were chiefly found in gene regions, and secondly in introns, this is consistent with previous findings on methylation in invertebrates [41, 42]. Among the many functional elements of genes, promoter methylation is widely recognized as a crucial mechanism in regulating gene expression and has been the focus of extensive research [43]. Traditionally, promoter hypermethylation is associated with gene silencing by blocking transcription initiation mechanisms [44]. However, with further research and technological developments, it has become clear that there are challenged to this understanding [24] contrary to the traditional understanding increasing evidence suggests that promoter hypermethylation now also appears to be associated with high transcriptional activity [45], a phenomenon that suggests that the relationship between promoter methylation and gene expression is complex.

**Fig. 8:**
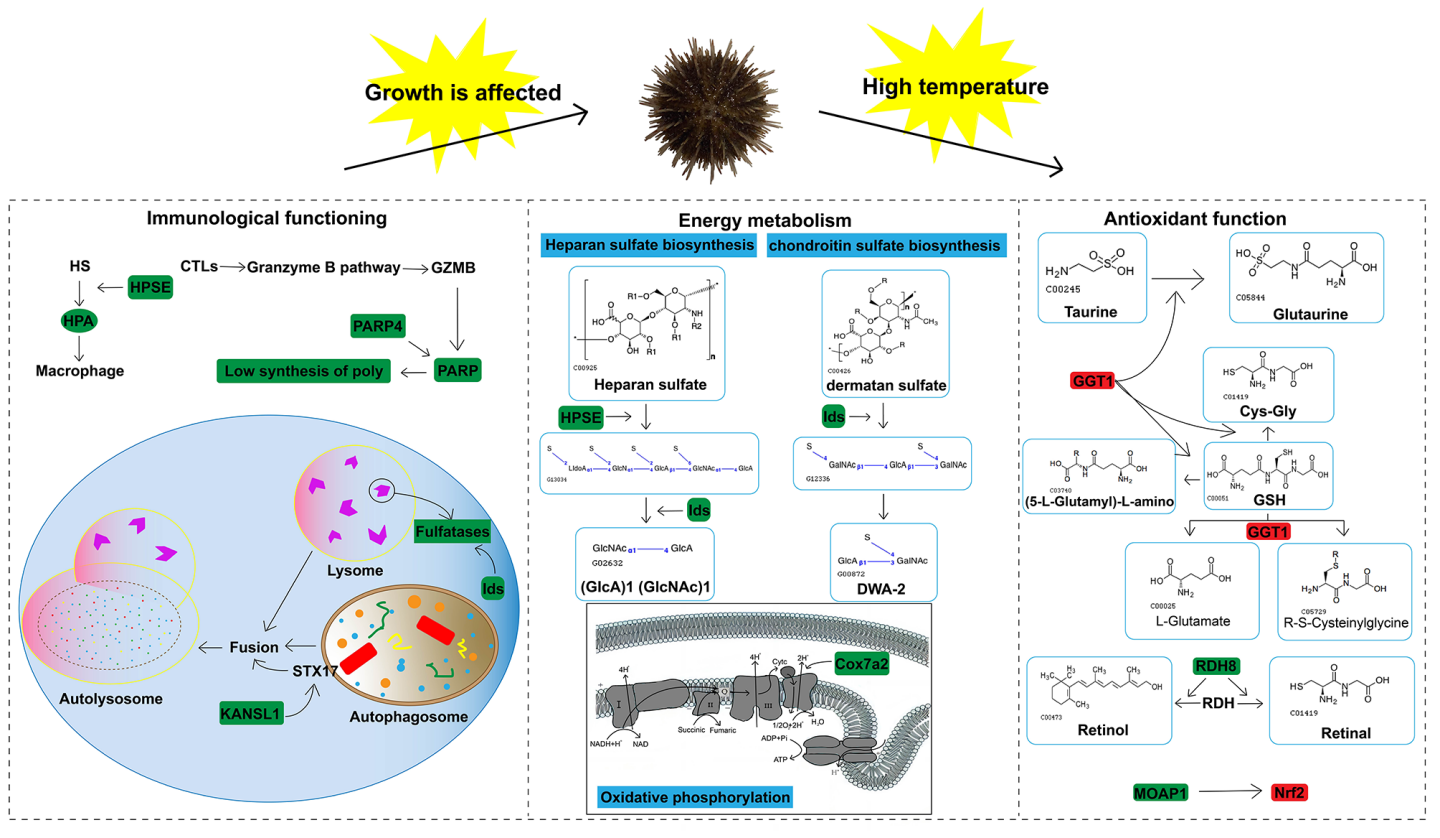
Diagram of the mechanism underlying the response of *Strongylocentrotus intermedius* to high temperature stress. Red represents upregulation/activation, and green represents downregulation/inhibition

Based on this complex relationship, we performed a joint analysis of promoter DMGs and DEGs. We found that 23 promoter DMGs were also DEGs, suggesting that these 23 genes may be regulated by DNA methylation. Some of these genes have been shown to play crucial roles in regulating immune function, energy metabolism, and antioxidant function, and thus affect the growth and development of the organism (Fig. 8):

*HPSE* encodes acetyl heparinase, which is associated with inflammatory processes in the body and can lead to extravasation of activated T lymphocytes [46]. The enzymatic substrate of acetyl heparinase is acetyl heparan sulfate (HS), a highly sulfated polysaccharide considered to have important biological functions [47–49]. HS can also form complexes with laminin, fibronectin, and collagen, thereby protecting the normal physiological function of the extracellular matrix (ECM) [49, 50]. HS exhibits various effects on the inflammatory response; including the ability to block extracellular mediators in vitro, regulate the interaction between leukocytes, endothelium and ECM, as well as interact with Toll-like receptors, inducing an innate immune response [51]. Consequently, the enzymatic response of acetyl heparinase to HS has a negative impact on the organism’s inflammatory response. Studies on mice have shown that overexpression of acetyl heparinase activates macrophages, and the cytokines secreted by activated macrophages stimulate endothelial cells to secrete acetyl heparinase, further enhancing macrophage activation and leading to inflammation [52].Environmental stress, such as high temperature, inevitably leads to inflammation. [53]. Previous studies have identified HPSE as a candidate marker for heat adaptation in vertebrates [54]. In this study, *HPSE* was found to be an overlapping gene between CG and CWG methylation pattern alteration genes. The upregulation of *HPSE* promoter methylation in *S. intermedius* along with a significant downregulation of gene expression under high temperature stress, indicates that the organism responds to inflammation caused by high temperature by decreasing the level of acetyl heparinase.

*KANSL1* regulates autophagosome–lysosome fusion through the transcriptional control of the autophagosome gene *STX17*. Lysosomes play a crucial role in the inflammatory response of cells and can bind to autophagosomes, which are tightly regulated during the process of autophagy. STX17 encodes an autophagosomal SNARE protein that is necessary for this fusion step [55]. While *STX17* is not upregulated during autophagy, its adequate expression is vital for the proper functioning of the autophagic process. Studies have shown that *KANSL1* is essential for autophagosome–lysosome fusion by binding to the promoters of autophagosome-related genes and regulating the transcription of *STX17* [56]. Numerous studies have demonstrated that high temperature stress can induce autophagy [57, 58]. In this study, the upregulation of *KANSL1* promoter methylation in *S. intermedius* was accompanied by a significant downregulation of gene expression under high temperature stress. *KANSL1*-mediated autophagy assists *S. intermedius* in clearing damaged mitochondria that are generated during the response to heat stress. Importantly, high temperature stress can also impair lysosomal function [59–61]. In this study, the gene associated with lysosomal enzymes, referred to as *Ids*, showed downregulation in promoter methylation, and its gene expression was decreased under high temperature stress, it also be an overlapping gene between CG and CWG methylation pattern alteration genes. *Ids* are responsible for encoding sulfatases, which is key enzymes involved in lysosomal function [62]. These findings indicate that the immune function of *S. intermedius* was affected by high temperature stress.

*Cox7a2* encodes a protein that serves as a subunit of cytochrome *c* oxidase (Cox). Cox is composed of 13 subunits, which are encoded by two genes [63].As the terminal component of the mitochondrial respiratory chain, Cox plays a crucial role in ATP synthesis [64]. In this study, the methylation level of the *Cox7a2* gene was significantly increased in the high temperature group. Consequently, the gene expression of *Cox7a2* was significantly decreased, leading to reduced synthesis of Cox. This reduction in Cox synthesis inhibited its activity, thereby impacting the energy metabolism efficiency of *S. intermedius*. Similar findings were observed in a study on *Procambarus clarkii*, it was found that the gene expression of the Cox subunit was significantly reduced under high temperature stress, resulting in an imbalance in energy metabolism [65]. Furthermore, *Cox7a2* expression was significantly downregulated. Another study on energy metabolism in *Portunus trituberculatus* also found that high temperature stress inhibited the expression of the *Cox* gene [66].

The *MOAP-1* gene regulates the Nrf2 signaling pathway, which is essential for protecting cells from oxidative stress. [67] Overactivation of Nrf2 signaling often occurs due to the excessive accumulation of p62/SQSTM1 vesicles that segregater Keap1, an adapter for the E3 ubiquitin ligase complex of Nrf2 [68]. Previous studies have reported that MOAP-1, a Bax-binding protein, can regulate the p62-Keap1-Nrf2 pathway. MOAP-1 can be recruited to p62 vesicles and independently downregulate its expression independently of the autophagic pathway during p62 vesicle production. Deletion of *MOAP-1* leads to a significant upregulation of p62 vesicles, enhanced isolation of Keap1 by p62, and overactivation of Nrf2 antioxidant target genes [69]. *MOAP-1* is closely associated with cell apoptosis [70]. When the body undergoes apoptosis due to environmental stimulation, *MOAP-1* is up-regulated [71]. High temperature stress, as an environmental stressor, can inevitably induce apoptosis and oxidative stress in the body [72]. However, in the present study, *MOAP-1* was downregulated. The cause of this phenomenon may be attributed to the effect of promoter DNA methylation. The downregulation of *MOAP-1* can reduce the apoptosis of the body and activate the Nrf2 signaling pathway to enhance the antioxidant function of the body.

In addition to this, the levels of certain antioxidant substances in the organism can also impact its antioxidant function. Glutathione is a commonly found antioxidant substance, and GGT1 plays a role in increasing intracellular glutathione levels by supplying cysteine to cells as part of the cellular antioxidant mechanism [73–75]. In this study, we observed an elevation in the promoter methylation level of *GGT1* which was correlated with an increase in its expression. The higher expression of *GGT1* contributed to the accumulation of glutathione in *S. intermedius*, thereby enhancing its antioxidant properties under heat stress. Retinol is another effective antioxidant substance that can also enhance the immunity of body. A study involving mice found that *RDH8* is capable of converting all-trans retinal to all-trans retinol [76–78],,and it also plays a vital role in aquatic animals [79, 80]. However, in this study, we observed a decrease in the methylation level of *RDH8*, resulting in reduced gene expression. This unfavorable change in *RDH8* levels may hinder the accumulation of retinol in *S. intermedius*, thereby potentially influencing its antioxidant function .

## Conclusion

In summary, the growth of *S. intermedius* is significantly correlated with temperature. Under high temperature stress, DNA methylation plays a role in regulating gene expression in *S. intermedius* and the relationship between promoter methylation and gene expression is not unique. High temperature stress induced substantial changes in DEGs and DMGs in *S. intermedius* cells. These genes were enriched in glycosaminoglycan degradation, oxidative phosphorylation, apoptosis, glutathione metabolism, arachidonic acid metabolism, thermogenesis, and others associated with phenotypic variation. These metabolic pathways affect immune function, energy metabolism, and antioxidant function, and ultimately affect growth and development of *S. intermedius*. The presented experimental data provide new insights into the epigenetic mechanisms underlying the adaptation of *S. intermedius* to high temperature stress. In the future, we will conduct more comprehensive studies to explore the relationship between other methylation positions and gene expression in *S. intermedius* under high temperature stress. Furthermore, we also intend to examine the impact of various stress conditions on DNA methylation in *S. intermedius*.

## Methods

### Growth experiment of *S. intermedius*

In the *S. intermedius* growth experiment, sea urchins with three different shell diameters (2 cm, 4 cm, and 6 cm) were cultivated at six different temperatures (10 °C, 14 °C, 18 °C, 22 °C, 24 °C, and 26 °C). Therefore, there were a total of 18 treatment groups, with 120 sea urchins in each group. The experimental period was 35d, and three parallel sets were set for each group.

*S. intermedius* cultivation was carried out in temperature-controlled tanks with circulation. Sea water was filtered by a sand filter at a salinity of 30 Formulated diets containing fresh *Ulva lactuca* were used, the feces was aspirated from the bottom of the tank every two days, and only one fifth of the water was changed to ensure that the temperature fluctuation was within 0.2 °C. The sea urchins were weighed once a week and the average weight gain rate (WGR) was calculated.

### High temperature stress experiment

*S. intermedius* with a shell diameter of 4 cm were selected for the high temperature stress experiment. According to the model prediction, the high temperature treatment group (24 °C) and the control group (15 °C) were set up. Each group included 60 healthy sea urchins. The experiment was conducted three times. A significant difference in body weight was observed after 35 days, and the experiment was stopped. Daily management was the same as in the growth experiment.

### Sample collection

At the end of the high temperature stress experiment, the sea urchins were dissected in a sterile fume hood. In total, 15 sea urchins from each group were selected. To reduce differences between individuals, the gut tissue of five sea urchins was pooled. The tissue was gently rinsed and cleaned with pure water. Gut samples for transcriptome and methylome assays were rapidly frozen in liquid nitrogen and stored at -80 °C until further analysis.

### MethylRAD analysis

DNA was extracted using the cetyltrimethylammonium bromide (CTAB) method. DNA quality, concentration, and integrity were examined using a nucleic acid quantifier (Thermo, NanoDrop 2000) and by 1% agarose gel electrophoresis. After quantification, DNA samples were stored at -80 °C. Libraries were constructed using MethylRAD-seq technology and high throughput sequencing was performed on an Illumina SE sequencing (50 bp) platform.

The raw sequencing data were filtered to obtain clean reads, which were compared to the reference genome using bowtie2 (version 2.3.4.1). The untranslated regions were annotated using SnpEff software (V4.1 g), and the distribution of methylation sites in different gene elements was determined using bedtools (V 2.25.0) (including the 2-kb region upstream of the TSS, the gene body, and the 2-kb region downstream of the transcription termination site [TTS]). The DESeq package (V 1.36.0) was used to screen for differentially methylated sites (DMSs) by normalizing the sequencing depth. The following threshold values were used: *P* < 0.05 and |log_2_(fold change)| > 1. The distribution of methylation sites in different genetic elements was determined using bed tools. The *P*-value and log_2_(fold change) of each locus were calculated using edgeR software. According to the sequencing depth of each locus in the six samples, methylation levels were compared between two groups; genes with *P* ≤ 0.05 and |log_2_(fold change) | > 1 were screened, their methylation levels were determined, and they were annotated. Finally, GO and KEGG enrichment analyses were performed on the differential genes.

### Total RNA extraction and transcriptome analysis

Total RNA was extracted using the mirVana miRNA Isolation Kit (Ambion, Texas, USA) following the manufacturer’s protocol. Total RNA quantity and integrity were assessed using the Agilent Bioanalyzer 2100 system (Agilent, CA, USA) and by 1% agarose gel electrophoresis [81]. The mRNA was enriched using oligo (dT) magnetic beads and fragmented to synthesize cDNA for PCR amplification. The cleaved RNA fragments were reverse-transcribed to create the final cDNA library following the protocol for the mRNA Seq sample preparation kit (Illumina, San Diego, USA) [82]. Raw data were filtered to obtain clean reads, which were mapped to the reference genome (not yet published) using HISAT software for further bioinformatics analysis [83].

Differential expression analysis of genes was performed using the DESeq R package (2012) and the negative binomial distribution (NB) test [84]. A p-value < 0.05 and Fold Change > 2 was set as the thresholds for significantly differential expression. Assembled transcripts were annotated using the KEGG and GO databases. Gene enrichment analysis was performed to identify (i) enriched GO terms in the biological process, cellular component, and molecular function categories and (ii) enriched biological pathways. Hypergeometric tests were performed to identify significantly enriched GO terms and KEGG pathways (*P* < 0.05) [85].

### Quantitative real-time PCR

To validate the RNA-seq results, we performed real-time fluorescence quantitative PCR (qRT-PCR). 18 S rRNA was used as the internal reference gene. Specific primers were designed using Primer Premier 5 (Table 6) and synthesized by Bio. Before experiments, gene-specific primers designed based on the assembled transcriptomes were firstly evaluated using the regular PCR method, A single PCR band was found with expected size by 1.0% agarose gel electrophoresis analysis. In this experiment, the gene-specific PCR amplification efficiency (E) ranged from 90 to 105%, and correlation coefficient (R^2^) greater than 0.98. primers possess excellent specificity and amplification efficiency. qRT-PCR was performed using a real-time qPCR system and SYBR Green. The PCR program was as follows: 95 °C for 30 s, 95 °C for 5 s, 60 °C for 32 s, 95 °C for 15 s, 60 °C for 60 s, 95 °C for 15 s, 95 °C for 15 s, and 60 °C for 15 s. Each PCR was performed in triplicate. Fold change values were calculated using the 2−ΔΔCt method.

**Table 6:** qRT-PCR primer design

Geng name	Forward primer sequence	Reverse primer sequence
*Cox7a*	TCTGAAGAAGGGAGGGATGGACTAC	GGCGTACTGGAAGAGAGCGAAAC
*PLXNA4*	AACTGTGGTTGGTGTGGTGATGAC	GGGCATTGATAGGAACTGACAAGGG
*Slc5a3*	GAATGATCCTCGACTTCGCCTACAG	GAGACTGACCACCACATTGACCAAG
*HPSE*	CAACACATCCACGGTCCTCAGAAC	GTTGTCCAAGCGTGCGATTGAAG
*SULT1B1*	CAGCAAGGCAGTCGGCAGATTC	AATTCAAGTCCAGATTCGCCCAGTC
*GGT1*	CGGTTGTCGTTGCTGTCCTGAG	AACACTGGACTTCAAATCGGTAGCG
*Orct*	GGACCTTCTAAGCGAGCCATTGC	ACGAGCAGAGTAGCAGGTAGTAAGG
*HSP70*	CGATGTGGCTCCTCTTTCTCTTGG	GATGGTAACGGCTGGCTGGTTG
*FOLH1*	TGATGGCTCTGAGGCGGTCTATC	TCCTCCTCCACTCCCAACCATTATG
*TMPRSS4*	TCCCATCTACAATCAGACGCAATGC	CAACACCGCCCTCTTTCAGTCC

### Statistical analysis

All data are expressed as the mean ± standard deviation. SPSS 22.0 software (SPSS, USA) was used for statistical analysis. We considered *P* < 0.05 as statistically significant and *P* < 0.01 as statistically extremely significant. The model was built according to the WGR data of sea urchins with different shell diameters at different temperatures. WGR was calculated as follows: WGR (%) = (W_t_ − W_0_)/W_0_ × 100%. Origin 2016 (OriginLab, USA) was used to establish the quadratic curve regression model, and Design expert 10.0 (State-East, USA) was used to establish the software response surface graph. The reliability of the model was analyzed based on the correction coefficient. Pearson correlation analysis was conducted to calculate the correlation between gene expression and DNA methylation based on transcriptome and DNA methylation sequencing data. A four-quadrant map was drawn, and GO and KEGG enrichment analysis was performed.

## Data Availability

The datasets presented in this study can be found in online repositories. The names of the repository and accession number can be found below: https://dataview.ncbi.nlm.nih.gov/object/PRJNA964456?reviewer=clji5ri98us3njv7tmgs6v3gs0.
